# Uncertainty Quantification and Flagging of Unreliable Predictions in Predicting Mass Spectrometry-Related Properties of Small Molecules Using Machine Learning

**DOI:** 10.3390/ijms252313077

**Published:** 2024-12-05

**Authors:** Dmitriy D. Matyushin, Ivan A. Burov, Anastasia Yu. Sholokhova

**Affiliations:** A.N. Frumkin Institute of Physical Chemistry and Electrochemistry, Russian Academy of Sciences, 31 Leninsky Prospect, GSP-1, 119071 Moscow, Russia

**Keywords:** retention time, retention index, collision cross-section, uncertainty estimation, deep learning, metabolomics

## Abstract

Mass spectral identification (in particular, in metabolomics) can be refined by comparing the observed and predicted properties of molecules, such as chromatographic retention. Significant advancements have been made in predicting these values using machine learning and deep learning. Usually, model predictions do not contain any indication of the possible error (uncertainty) or only one criterion is used for this purpose. The spread of predictions of several models included in the ensemble, and the molecular similarity of the considered molecule and the most “similar” molecule from the training set, are values that allow us to estimate the uncertainty. The Euclidean distance between vectors, calculated based on real-valued molecular descriptors, can be used for the assessment of molecular similarity. Another factor indicating uncertainty is the molecule’s belonging to one of the clusters (data set clustering). Together, all three factors can be used as features for the uncertainty assessment model. Classification models that predict whether a prediction belongs to the worst 15% were obtained. The area under the receiver operating curve value is in the range of 0.73–0.82 for the considered tasks: the prediction of retention indices in gas chromatography, retention times in liquid chromatography, and collision cross-sections in ion mobility spectroscopy.

## 1. Introduction

**Figure 1:**
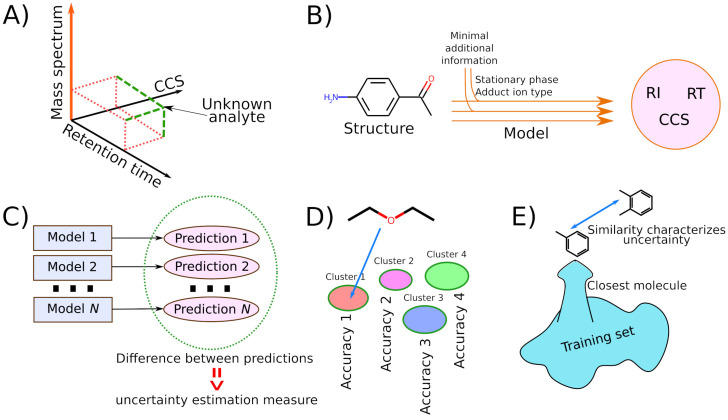
(**A**) Illustration of how knowledge of several factors (mass spectrum, retention time, ion mobility spectroscopy data) helps to identify an unknown analyte; (**B**) schematic depiction of predicting the molecule property from the structure; (**C**) the spread of predictions of several models forming an ensemble can be used as a measure of uncertainty; (**D**) different accuracies of predictive models in different clusters into which the chemical space is divided; (**E**) the molecular similarity between the closest molecule from the training set and the molecule for which the prediction is made characterizes the uncertainty.

In recent years, machine learning and deep learning (DL) have been increasingly used in analytical chemistry and metabolomics [1,2,3,4,5], in particular, for tasks such as predicting retention indices [1,6,7,8,9] (RI) in gas chromatography (GC), retention times [3,10,11] (RT) in high-performance liquid chromatography (HPLC), and collision cross-sections [2,12,13] (CCS) in ion mobility spectroscopy (IMS). These methods, GC, HPLC, and IMS, are used in metabolomic analyses, along with mass spectrometry, to determine the structure of unknown compounds. For each unknown substance, as a result of a mass spectrometric experiment, there is information on its molecular weight, the mass spectrum formed as a result of fragmentation in the ion source (e.g., electron ionization) or in the collision cell, as well as one or more additional numerical characteristics: RI, RT, and CCS. The basis for identification is the mass spectrum; identification can be made using a mass spectral database [14,15] or using artificial intelligence methods [16]. Unfortunately, identification based on the mass spectrum is very inaccurate and often leads to an incorrect result [15], so it is important to use RI, RT, and CCS as an additional (orthogonal) characteristic to discard incorrect candidates and refine identifications (Figure 1A). The reference databases are not large and the prediction of these characteristics, using machine learning based on the structure of the molecule (Figure 1B), is helpful.

Usually, when neural networks (NN) [7,8], regression tree-based gradient boosting (GB) [11], and many other machine learning methods [9] are used, the output model provides only one number (the predicted property of the molecule) without any uncertainty estimation (UE). The model’s accuracy is then assessed using a large data set containing many different molecules, and the reliability of each specific prediction remains unknown. Then, in mass spectral identification, a threshold approach is often used [2,11,17]. In this case, candidates with a discrepancy between the observed and predicted values greater than a predetermined threshold are discarded. This approach can lead to completely meaningless results when the data set, on which the accuracy was assessed, and the molecules, for which the analysis was performed, belong to different statistical populations. It is thus necessary to assess the uncertainty of each prediction.

There are several approaches to UE in DL [18,19,20,21]. Such approaches include dropout, Bayesian neural networks (BNN), and ensembles of several models. These three groups of methods (each has many variations [18]) are versatile and are used in a wide range of scientific fields [22,23]. However, the first two methods impose requirements on the architecture, can only be applied to models of a certain type, and cannot be applied to already trained (and published) models. In addition, a number of studies have indicated that of these methods, ensembles have the best ability [19,20,21] to estimate UE. Also, in addition to UE, ensembles out-of-the-box allow for achieving the highest prediction accuracy and can use different types of models simultaneously. Thus, of these three approaches (BNN, ensembles, and dropout), the use of ensembles is preferred as it is the most versatile, giving the most accurate predictions [6,7,8,24], and the most reliable [19,20,21] UE. In the present work, we leave dropout- and BNN-based methods out of the scope of the study.

A chemistry-specific approach to UE is the use of molecular similarity [3,9,25,26]. The greater the similarity between the molecule under consideration and the closest molecule from the training set, the more accurate the prediction is likely to be [9,26]. Approaches based on molecular similarity have been used for rough UE, in predicting chromatographic retention [3,9,26]. Molecular similarity (a quantitative measure of the “similarity” of two molecules) can be calculated by different methods; the most computationally efficient and frequently used method is to compare two molecular fingerprints (MF) [27,28], i.e., binary vectors characterizing the presence or absence of certain features in a molecule. There are different methods for calculating MF [27]. It is also possible to estimate molecular similarity by finding the maximum common substructure (MCS) between two molecules [29]. Other approaches, for example, comparing real-valued molecular descriptors (MD), can be also used.

Previously, mostly for illustrative purposes rather than for UE purposes, it has been shown that the accuracy of predicting molecular properties for the same model varies significantly depending on which class the molecule belongs to [7,8,30,31]. There are numerous automated methods for classifying molecules based on their structures [32]. Most of these approaches include hard-coded rules and heuristics to assign a molecule to one of the understandable and chemically meaningful classes. However, in the case of polyfunctional, unusual molecules, one cannot be sure that such a classification will yield meaningful results. Therefore, instead of chemically meaningful classification methods, we decided to use, in this work, unsupervised machine learning to split a set of molecules into clusters.

To the best of our knowledge, there are no studies in which comprehensive models using both general and chemistry-related features were used for UE. Of the general approaches, we considered the one that is the most versatile and accurate. The aim of this work is to compare three approaches to UE in predicting mass spectrometry-related properties of molecules and to develop a model for UE that uses all three factors at once. These approaches are based on ensembles of models (Figure 1C), the clustering of a set of molecules (Figure 1D), and molecular similarity (between the molecule under consideration and molecules from the training set) (Figure 1E). The obtained results can be used in mass spectrometric analyses of metabolites and will increase the accuracy of tentative identification in metabolomics.

## 2. Results and Discussion

### 2.1. The Prediction Tasks Considered and the Study Design

The present study considers four problems of predicting mass spectrometry-related properties of molecules based on their structure: RI for polar and non-polar stationary phases, RT under reversed-phase HPLC conditions, and CCS. Non-polar stationary phases in GC are stationary phases based on polydimethylsiloxane and 5%-phenyl polymethylsiloxane (e.g., DB-1, DB-5). Polar stationary phases are stationary phases based on polyethyleneglycol (e.g., DB-WAX). All tasks (with the corresponding designations) and information about the corresponding data sets are presented in Table 1. The size and diversity of the data set affect the accuracy of the predictive model itself. The larger and more diverse the data set, the more accurate the predictive model. Therefore, it certainly makes sense to use as large and diverse data sets as possible.

In this study, we considered the absolute error rather than the relative error. The values under consideration are far from 0. For example, all retention times for the RT task are in the range of 500–1500. For the RI_NP task, 98% of retention index values are in the range of 719–3578. Thus, the values of the relative error corresponding to the same absolute error differ by no more than several times (less than an order of magnitude). Since UE is performed for a specific molecule, absolute uncertainty can easily be converted to relative uncertainty.

The values of accuracy measures (cross-validation) for the corresponding ensembles of models are also given there. Accuracy measures are given for the full ensembles. Neural networks of various types, support vector regression (SVR), and GB were used. For all tasks except RI_NP, the ensemble prediction is the average of the predictions of the models in it. For RI_NP, a linear combination of the models with unequal coefficients is used; details are given in the corresponding publication [7]. For RT and CCS, the corresponding values of MAE and MdAE, the corresponding differences between model predictions, etc., are written in seconds and Å2, respectively, throughout this study. The dimension is omitted to improve readability and unify the presentation of data for different tasks.

The mean absolute error (MAE) and median absolute error (MdAE) were used as accuracy measures. For the RI_WAX and RI_NP tasks, the provided MAE and MdAE values are higher than those reported in the previous publications [7,8], despite the use of the same data set and the same models. This is due to the fact that in those works [7,8], many RI values were considered for some molecules during the calculation, which increased the statistical “weight” of well-studied and usually better-predicted molecules. In the present study, exactly one experimental and predicted value is considered for each molecule (see the Section 3) in all cases. When forming the data sets used in this work, we did away with the overrepresentation of some molecules; each molecule is represented only once.

For each task, the experiment was conducted as follows: the data set was split into 10 (RI_NP) or 5 (other tasks) parts, so that all records for each of the molecules were assigned to one of the subsets. Then, for each subset, a prediction was made using each of the models trained using all other subsets as a training-validation set. This procedure is essentially the same as regular cross-validation but is used to construct a data set rather than simply assess the accuracy of models. As a result, we obtain predictions (using each of the models) for each of the molecules from the entire data set. The predictions were made in such a way that the given molecule was not in the training set when training the corresponding predictive models. This allows us to avoid any bias associated with overfitting. Thus, the result is a data set with each row containing the structure of the molecule (in the form of a SMILES string), the predictions made using a number of models, as well as the number of the subset to which it belonged. If two molecules belonged to different subsets, this meant that each of them was in the training set for the models that made predictions for the other of them.

Our goal in this work is to demonstrate that our approach is model-agnostic and works for different types of models. In all cases, we took models with an accuracy close to the state-of-the-art, but we deliberately chose the tasks and models to demonstrate the versatility of our approach and its applicability to different models. For the RI_WAX task, the models used in this study [8] are the most accurate to date and have state-of-the-art accuracy to the best of our knowledge. For the CCS task, the most commonly used models are usually based on support vector regression and other shallow learning methods [2,30]. The graph neural network allows us to achieve a very small increase in accuracy, but at the expense of using an overly complex model [13]. It is important to note that for the CCS task, the ensemble consists of 10 SVR models. Unlike neural networks, SVR models are quite deterministic and even varying hyperparameters does not allow us to achieve a spread of predictions using the same data set. In this case (as well as for the RT task), we used bootstrapping, removing ~10% of molecules directly from the training set, different for each of the models included in the ensemble. This is important when using SVR; otherwise, the errors produced by the models will be too correlated.

Then, based on this data set, the calculation of features was performed. The features characterize (i) how similar, in structure, a given molecule is to the molecules included in the training data set, (ii) how close the predictions of different models are to each other, (iii) which of the clusters a given molecule belonged to and how far from the cluster centroid it is. To calculate the Euclidean distance and for clustering, the principal component analysis (PCA) method was used. We considered 20 principal components of the vector of 2D MD. Clustering was performed using the *k*-means method. A detailed description of the training procedure for each of the models, the frameworks and MD used, the molecular similarity algorithms, and all other approaches used are given below in the Section 3. The obtained features and the corresponding designations are given in Table 2. The rest of this work is devoted to how these features allow us to perform UE.

In this study, we investigated the use of each of the features listed in Table 2 as a value that shows whether a prediction can be considered satisfactory. The threshold indicating which prediction can be considered satisfactory was chosen for each task so that 15% of the predictions in the corresponding data set were satisfactory. It was shown that the best results are achieved when using features *M*_s1_ or *M*_s2_ as a predictor. This is consistent with the results of previous works showing [19,20,21] that this is the approach that is well-suited for UE. The features *S*_max, e_ and *S*_max, t_ also work well. The features related to clusters work much worse when used as the only predictor. However, it is possible to significantly improve the accuracy of UE if a linear combination of several features is used simultaneously (logistic regression). This allows us to increase the accuracy of UE, compared to using only the *M*_s2_ feature. Importantly, the use of this feature (the spread of predictions of the models included in the ensemble) is the most accurate and versatile UE method [20,21] that does not use chemistry-related features. The values of the F1-score accuracy measure for all four tasks and various classifiers are given in the Supplementary Materials Section S1. The values of other accuracy measures and a detailed discussion of each approach are given below.

### 2.2. Assessment of the Reliability of the Uncertainty Estimation

**Figure 2:**
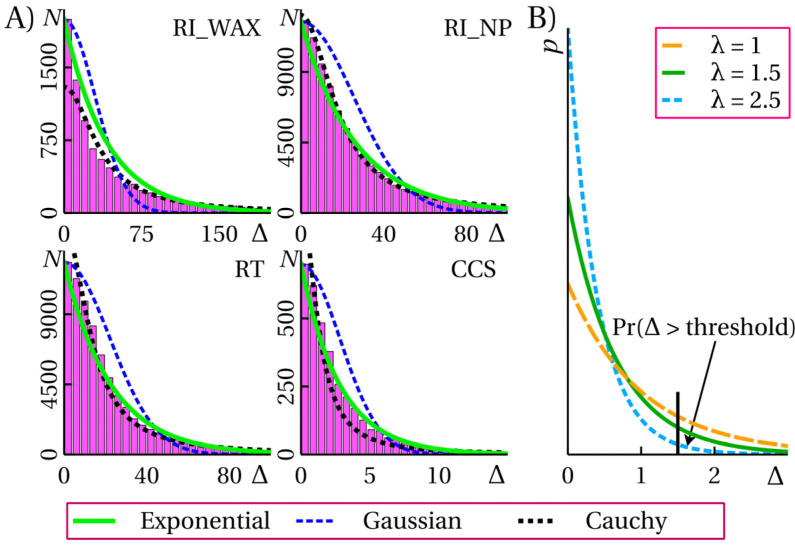
(**A**) Distributions of absolute prediction errors Δ of the considered ensemble models; (**B**) the probability that Δ is greater than the given threshold value is uniquely determined by the parameter λ of the exponential distribution.

Many studies [1,6,33] assume that the errors of the predictive models are normally distributed. However, for the models and tasks considered in this study, the error distribution over the data set is much closer in shape to an exponential distribution than to a normal one (Figure 2A). For some of the tasks, there is a good match between the observed distribution and the Cauchy distribution, which does not have a finite variance. A similar error distribution was observed in a number of previous studies [34,35]. The shape of the error distribution may not be so important if the researcher aims only to achieve the best prediction accuracy. However, when assessing uncertainties, the way the errors are distributed is important. In many cases, UE algorithms predict the standard deviation [6,33] for the predicted value. In this case, an analytical chemist or a developer of the mass spectral identification software may assume that the error is distributed according to a normal distribution, the variance of which is predicted by the UE algorithm. This will lead to a significant underestimation of the probability of large (more than 2 sigma) errors if the actual shape of the error distribution is exponential (tailed). Thus, predicting the standard deviation as a result of the UE algorithm may be misleading. Strictly speaking, the true shape of the prediction error distribution for a particular molecule (the prediction error is a random variable) is unknown and there is no reason to believe that, for sophisticated machine learning methods, this quantity is normally distributed.

In this study, we also consider an alternative formulation of the UE problem. Instead of predicting (for each specific molecule) the real-valued uncertainty (standard deviation, confidence interval), we consider predicting the probability that the error is greater than some threshold. If the error is a random variable whose distribution is given by a single parameter (e.g., a normal distribution or an exponential distribution), then both formulations are equivalent. The probability that the error is greater than some threshold is unambiguously related to the variance of the distribution (Figure 2B). Since predictions with very large uncertainties are virtually useless for chemical analysis, predicting that the error is likely to be greater than the threshold is convenient for software design. Such a prediction is red-flagged and is not included in the candidate scoring.

The error threshold value, below which the prediction is considered satisfactory, is designated as *D_t_*. The *D_t_* values corresponding to 80, 85, 90, and 95% of satisfactory predictions in the data set were considered. The corresponding *D_t_* values are given in Table 3. In this case, to assess the accuracy of the UE algorithm, one can use the measures used to assess the accuracy of classification algorithms: recall, precision, etc. As the primary measure, we use the area under the receiver operating characteristic curve (ROC-AUC).

In the previous work [17], the threshold values for rejecting incorrect candidates in GC-MS identification were 70 and 100 for non-polar and polar stationary phases, respectively. The *D_t_* values corresponding to a small fraction of “unsatisfactory predictions” in the data set are too large for practical use. In most cases (unless otherwise specified), we use the value corresponding to 15% of unsatisfactory predictions in the data set as the *D_t_* value.

### 2.3. Using Molecular Similarity for Uncertainty Estimation

The first approach to UE considered in the present study is the approach based on molecular similarity. There are two possible ways in which molecular similarity can be used for UE. The first idea is based on using the fraction (or number) of molecules similar to the molecule under consideration in the data set as a measure. The term “similar” refers to molecules for which the molecular similarity measure values are not lower than a certain threshold value. The other approach [3,9,26] is to use the *S*_max_ value, which is the molecular similarity measure between the molecule under consideration and the molecule from the training set for which this measure is a maximum. This approach has an important advantage—the absence of hyperparameters to be chosen (the similarity threshold value should be selected). For simplicity, we used only the second approach (based on the *S*_max_ value). Preliminary experiments showed that using the first approach does not provide a significant advantage.

**Figure 3:**
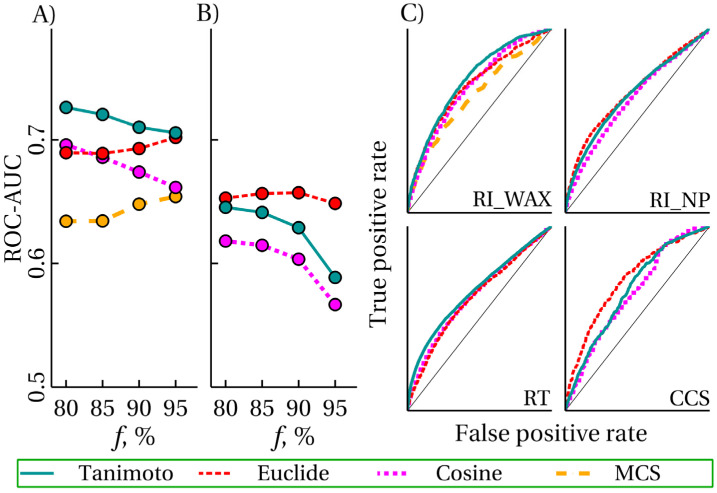
(**A**,**B**) Dependence of the accuracy measure ROC-AUC (area under the curve) of the classifier that predicts whether a prediction is unsatisfactory on the threshold value indicating what is considered unsatisfactory; the threshold value is set by the fraction *f* of satisfactory predictions in the data set; data are given for the tasks RI_WAX (**A**) and RI_NP (**B**); the classifier is based on various molecular similarity measures; (**C**) receiver operating characteristic curves for different tasks and classifiers based on different molecular similarity measures; data are given for *f* = 85%.

Four molecular similarity measures were considered: the measures based on the Tanimoto similarity of MF, the cosine similarity of MF, the Euclidean distance of principal components of vectors composed of MD, and MCS. These molecular similarity measures correspond to the *S*_max, t_, *S*_max, c_, *S*_max, e_, and *S*_max, mcs_ values (see Table 2), respectively. Figure 3 shows the ROC-AUC values and the receiver-operating curves for different *D_t_* values, different tasks, and different molecular similarity measures. In constructing this figure, the *S*_max_ values were used as predictor values for the binary classification task (is prediction satisfactory or not). In this case, the *D_t_* values correspond to 80, 85, 90, and 95% of satisfactory predictions in the data set (see Table 3). It is evident that the MCS algorithm performs significantly worse than other algorithms. In addition, this algorithm is 1–2 orders of magnitude slower than others, and existing implementations contain bugs. So, for a number of pairs of molecules, RDKit works for such a long time that, without using a timeout, it is almost impossible to achieve a result. Therefore, MCS was only applied to the RI_WAX task and was not applied to other data sets.

The algorithm based on Euclidean similarity is the only one whose accuracy does not decrease with the increasing threshold value *D_t_* of the remaining algorithms. In most cases, it is either the most accurate or the second most accurate. Molecular similarity based on binary MF is most commonly used for UE [3,9,26]. The present study demonstrates that the Euclidean similarity of principal components of vectors composed of real-valued MD performs not worse or even better.

**Figure 4:**
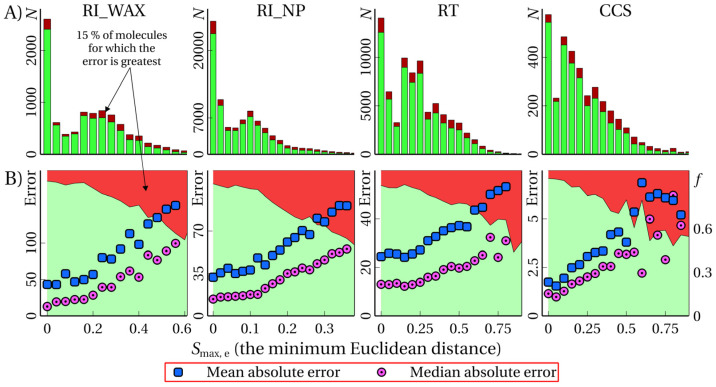
(**A**) Distribution of the number *N* of molecules by the *S*_max, e_ values (the smallest Euclidean distance from a molecule to molecules from the training set); dark red color shows unsatisfactory predictions; (**B**) dependence (the boundary between the red and light green areas) of the fraction *f* of unsatisfactory predictions, the mean and median absolute errors (in bins) on the *S*_max_ value; in both (**A**) and (**B**), the data are given for different tasks (marked at the top).

Figure 4 shows the distribution of satisfactory and unsatisfactory predictions by *S*_max, e_ values for all tasks, and the *D_t_* values corresponding to 15% of unsatisfactory predictions in the data set. It is evident that the *S*_max, e_ value correlates with MAE and MdAE in the corresponding bin, as well as with the fraction of unsatisfactory predictions in the corresponding bin. Thus, the *S*_max, e_ value can be used as a predictor for UE. The values of the ROC-AUC measure (for the *D_t_* value corresponding to 15% of unsatisfactory predictions in the data set) for various tasks and various molecular similarity measures are given in Table 4. A similar table for the F1-score measure is provided in the Supplementary Materials Section S1. Examples of recall-precision curves are given in the Supplementary Materials Section S2. The dependence of the relative error on *S*_max, e_ for the RI_NP task is shown in the figure in the Supplementary Materials Section S3.

### 2.4. Spread Between Predictions of Different Models for Uncertainty Estimation

The most common and natural approach to UE is the approach based on the spread of predictions of the models included in the ensemble [20,21]. This approach has been repeatedly considered in the literature and has been used, among other applications, in the RI prediction [6]. In this case, the tasks RI_WAX and RI_NP, on one hand, and RT and CCS, on the other, differ significantly from each other. In the case of RI_WAX and RI_NP, the models included in the ensemble are significantly different and use different representations of molecules. In the case of RT and CCS, the models are the same. In the case of RT, all models are graph neural networks. In the case of CCS, all models are SVR, i.e., it is a typical example of nonparametric models. We deliberately selected the tasks in such a way to consider dissimilar situations.

It is obvious that such an approach allows for the predicting of the uncertainty associated with the uncertainty in the values of the model parameters and the uncertainty associated with the fact that the learning procedure is stochastic, and this introduces a random error. It is less obvious (although confirmed by previous works [20]) that the epistemic uncertainty associated with the fundamental inability of the used model to describe physical phenomena can be quantified in this way. In the case of using models of the same type in an ensemble, one can expect a situation where a model of a given type, for some reason, will give a significant error with the same sign for the same compound. A quantitative assessment of this phenomenon is outside the scope of this study.

**Figure 5:**
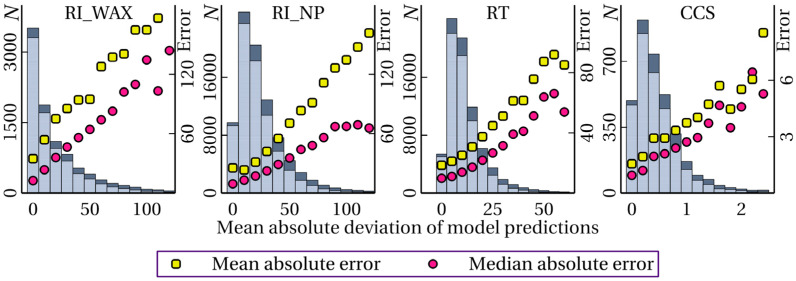
Distribution of the number *N* of molecules by the mean absolute deviation of model predictions (in the ensemble); dependence of the mean and median absolute errors in bins on the mean absolute deviation of model predictions; dark blue color shows unsatisfactory predictions; the data are given for different tasks (marked at the top).

Table 4 shows the ROC-AUC values obtained when *M*_s1_ and *M*_s2_ are used as predictor values, indicating the reliability of the prediction. The corresponding F1-score values are given in the Supplementary Materials Section S1. It is evident that these predictor values allow for achieving higher ROC-AUC values compared to other predictors, and therefore, a more accurate UE. However, it is also evident that the ROC-AUC value is still very small, that is, in a significant number of cases, this approach does not allow us to correctly determine whether the prediction is satisfactorily accurate. Figure 5 shows the distribution of the number of satisfactory and unsatisfactory predictions depending on the spread between the model predictions (*M*_s1_). The mean and median errors are also shown for each bin. It is clearly seen that, on one hand, the *M*_s1_ value can be used as a predictor for UE, and on the other hand, even the very first bins contain a significant fraction of unsatisfactory predictions. Thus, despite the fact that *M*_s1_ is a relatively well-performing predictor, the task of increasing the accuracy of UE is relevant. The dependence of the relative error on *M*_s1_ for the RI_NP task is shown in the figure in the Supplementary Materials Section S3.

### 2.5. Clustering for Uncertainty Estimation

**Figure 6:**
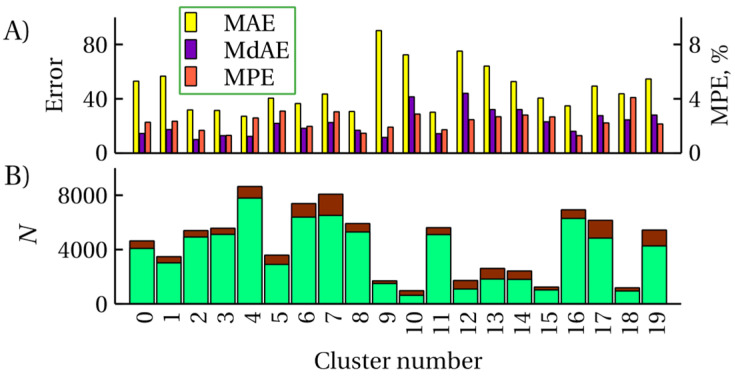
(**A**) The mean and median absolute prediction error as well as mean percentage error (MPE) in different clusters; the data are given for the RI_NP task; (**B**) distribution of the number *N* of molecules across clusters; dark red color shows unsatisfactory predictions.

A third possible approach to UE is to cluster the data set and take into account the fact that the MAE/MdAE and the fraction of unsatisfactory predictions vary significantly across clusters. Such data are presented for the RI_NP task in Figure 6. It should be noted that *k*-means clustering is a stochastic process and depending on the random choice of the initial centroid positions, the exact assignment of molecules to clusters, and hence, the accuracy for each cluster, will be different; Figure 6 shows an example of such a distribution. The assignment of a molecule to one of the clusters can be a criterion for UE. The predictive model for which UE is performed must be trained before this, and the accuracy measures of this predictive model must be calculated separately for each cluster.

In addition to UE, clustering the data set (and assigning a molecule to one of the clusters for which a prediction is made) can be useful for many other reasons. For example, the accuracy can be improved by clustering followed by training separate models for each cluster [36]. This approach is beyond the scope of this study.

**Figure 7:**
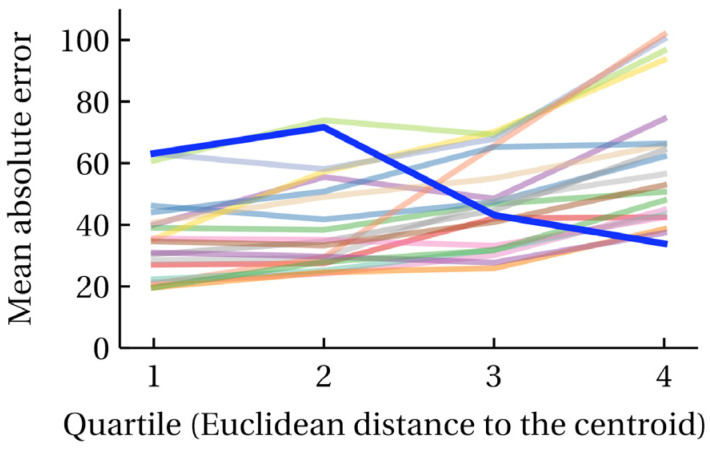
Dependence of the mean absolute prediction error on the distance to the cluster centroid (Euclidean distance, 20 principal components); the mean absolute error is given by distance quartiles for each of the clusters; each colored line corresponds to the dependence for one of the clusters; since clustering is a stochastic process, specific cluster numbers have no physical meaning; cluster 0 is highlighted in bold; the data are given for the RI_NP task.

It can be expected that, on average, molecules located closer to the cluster centroid are more “typical” and “simple”, while those located on the cluster periphery are polyfunctional and less typical. From these qualitative considerations, it can be expected that, on average, the prediction for molecules located closer to the centroid is more reliable. Molecules furthest from the centroid can essentially be regarded as outliers, randomly assigned to one of the clusters. Unlike other clustering methods, *k*-means does not prevent this in any way. However, in our case, we turn this disadvantage into an advantage; the distance from the cluster centroid is an additional feature characterizing the accuracy of the prediction. For the RI_NP task, for each cluster, the molecules were sorted and split into quartiles by their distance to the centroid, and the MAE was calculated for each quartile. These data are shown in Figure 7. It is evident that for all clusters except one (it has the number 0, but the cluster numbering is arbitrary and has no physical meaning), the prediction error increases with the distance from the centroid. What causes the anomalous behavior of cluster 0 remains unexplored. When re-clustering, the cluster numbers take on a completely different meaning, and the exact distribution of molecules across clusters also changes, but this behavior is often observed for some clusters.

**Figure 8:**
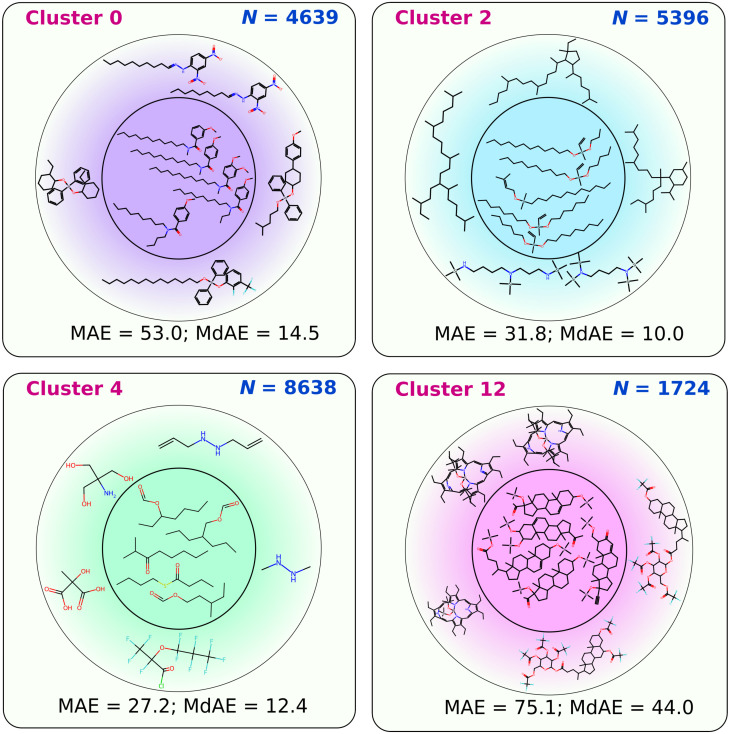
Examples of molecules from some of the clusters; the inner circle shows molecules that are close to the cluster centroid and the outer circle shows some of the molecules that are farthest from the cluster centroid; the data are given for the RI_NP task.

Figure 8 shows examples (RI_NP task) of clusters. The inner circle shows molecules close to the cluster centroid, and the outer circle shows molecules farthest from the centroid. Clearly, clustering using the *k*-means algorithm is an effective method for classifying the molecules included in the data set.

### 2.6. Comprehensive Models for Uncertainty Estimation

It was shown that there are three different approaches that provide comparable accuracy that can be used for UE: approaches based on molecular similarity, clustering, and similarity of predictions. Each approach calculates one or more real numbers, each of which can be used as a predictor, i.e., directly converted to uncertainty or the probability of incorrect prediction. Table 4 contains the ROC-AUC measure values for each of these values used as a predictor.

Since the approaches are very simple (the predictor values are calculated in a deterministic way, and there are no fitted parameters), it is natural to consider a machine learning model that takes into account all of the factors simultaneously (uses these factors as features). Common logistic regression can significantly improve the accuracy of UE. The corresponding values of the ROC-AUC measure are given in Table 4. Table 5 shows the features used for logistic regression. The logistic regression coefficients and corresponding standard deviations are also shown. Table 6 shows the confusion matrix and other accuracy measures for the obtained models. For the confusion matrix and measures working with binary values, the probability threshold value was chosen as *p* = 0.15, which approximately corresponds to the maximum of the F1-score accuracy measure. All data are given for the *D_t_* threshold values corresponding to 15% of the unsatisfactory predictions in the data set. The recall-precision curves are shown in the Supplementary Materials Section S2.

We have ensured that for the considered data sets, logistic regression is almost not subject to overfitting; the difference between the ROC-AUC for the training set and the test set (with random splitting of the data set) does not exceed 0.02 in all cases. For all tasks except CCS, this difference does not exceed 0.002. Therefore, in Table 5 and Table 6, for simplicity, we present data for the model trained and tested on the entire data set without cross-validation. The absence of observed overfitting is quite expected; the models contain only six fitted parameters and were trained on a data set containing thousands of records.

Some of the coefficient values in Table 5 are not statistically significantly and deviate from 0, but these coefficients are still included (with standard deviations) for completeness. Most of the coefficients are statistically significant with *p* > 0.999. Table 6 shows that among the molecules for which logistic regression predicts that the prediction is satisfactory, the fraction of unsatisfactory predictions is relatively small. For example, for the RI_WAX task, this fraction is only 5.8%, while in the whole data set it is 15%. Accordingly, this classifier (even if it does not work perfectly) can be used in a software that predicts the properties of molecules in order to flag obviously unreliable predictions. The MAE values differ significantly for the groups of molecules that the logistic regression labels as satisfactory and unsatisfactory. For the first group, the accuracy is quite good, as expected. The corresponding data are shown in Table 7.

Further improvement of UE accuracy is possible by using more complex, non-linear machine learning models. In such problems, the machine learning algorithm that gives the most accurate results and its hyperparameters are usually determined by trial and error. To avoid trying different machine learning algorithms manually, we used the H2o AutoML package [37], which “tries” different machine learning algorithms and different hyperparameters in a fully automatic mode and selects the most accurate one. The enumeration is carried out without human participation and, in general, the achieved accuracy is at the level that can be achieved manually [37]. ROC-AUC was used as a measure for comparing and selecting models, and a total of 100 model selection attempts were performed. All of the features presented in Table 2 were used as a set of features, the cluster number was one-hot encoded, and the prediction of the ensemble was also added as an additional feature. The results are shown in Table 4 and Table 8. The accuracy is determined using 5-fold cross-validation, the prediction threshold value for the classifier was selected automatically based on the maximization of the F1-score value. It is evident that the use of modern machine learning methods allows for achieving a slightly higher accuracy compared to the linear model. In all cases, models based on GB were selected.

**Figure 9:**
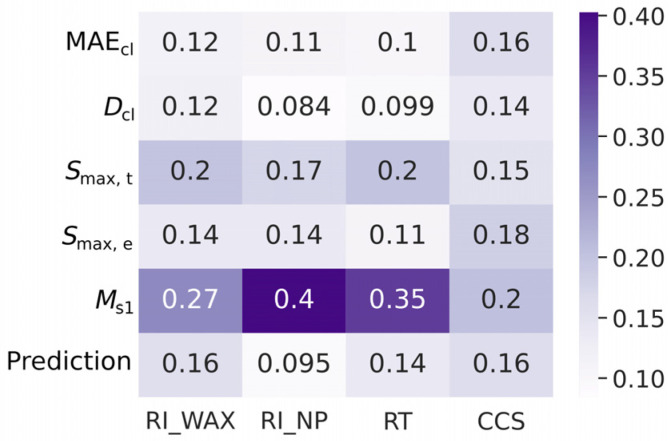
Feature importance (gain-based) in predicting the probability of a prediction being unsatisfactory using the gradient boosting.

We studied the importance of various features in UE using GB. The XGBoost package [38] was used. The default hyperparameters were used (max_depth = 3; learning_rate = 0.1; n_estimators = 100). The gain was used as a measure of feature importance. It shows the average gain across all splits where the feature was used. Of the groups of strongly correlated features whose physical meaning is close to each other (for example, MAE_cl_ and MdAE_cl_), only one was left. The results of the feature importance assessment are shown in Figure 9. It is evident that the *M*_s1_ feature is of the greatest significance. However, UE based only on the *M*_s1_ feature is significantly less reliable compared to using all the features considered (see Table 4), and such comprehensive models should be used when developing software for predicting the mass spectrometry-related properties of molecules.

The significance of some of these features is quite difficult to interpret meaningfully. The model is essentially an ensemble (kind of a meta-learner), and each of the main features (for example, mean absolute deviation of model predictions) is directly related to the predicted characteristic. The importance of features is directly related to the predictive ability of these features. Figure 9 shows that clustering does not contribute much to UE for all tasks except CCS. It is for CCS that the advantage of the *M*_s1_ feature over other features is minimal. This is probably due to the fact that this is the only task where deep learning was not used and the spread of models was provided mainly by bootstrapping (and varying hyperparameters). This is consistent with the data presented in Table 4 and in the Supplementary Materials Sections S1 and S2. The significance of the “ensemble prediction” feature is related to the fact that for large values, the absolute prediction error is, on average, larger.

Thus, the features listed in Table 2 can be used to roughly estimate the uncertainty of molecular property predictions. These features work for a wide variety of tasks and for a wide variety of models, both deep learning-based and models such as SVR. The spread between model predictions is the best predictor of UE among these features. However, it is their combined use that allows us to achieve the most accurate UE. This approach was first applied in this work. Gradient boosting provides a very small advantage over logistic regression. A comparison of accuracy measures for all models and predictors is given in Table 4 and in the Supplementary Materials Section S1. How MAE and MdAE depend on feature values is shown in Figure 4, Figure 5 and Figure 6. Table 7 illustrates how MAE differs for those predictions that logistic regression marks as satisfactory and unsatisfactory. A two- to three-fold difference in MAE is observed. Of the algorithms for calculating molecular similarity, the Euclidean distance (20 principal components calculated from molecular descriptors) is the best fit, as is the Tanimoto metric between molecular fingerprints.

## 3. Materials and Methods

### 3.1. Software and Frameworks Used

For chemoinformatics tasks (generation of MD, MF, molecular graphs, and atom-wise features) the CDK [39] (Chemistry Development Kit) framework, version 2.7.1, was used. The training of models for predicting gas chromatographic RI was performed exactly as described in our previous works [7,8] (using the Deeplearning4j [40] framework, version 1.0.0-beta6; and the XGBoost [38] library, version 1.0.0). More detailed information is given in the previous works [7,8]. The training of models for predicting RT (HPLC) was performed using the Julia [41] software (version 1.10.4) and the Flux [42] framework (version 0.14.18). The LIBSVM [43] library (version 3.25) was used to train models for predicting CCS. The Smile [44] framework (version 3.1.1) was used for PCA and clustering. The RDKit [45] framework, version 22.03.5, was used to find the MCS of two molecules. The molecules were stored as SMILES [46] strings, without using symbols denoting stereochemistry. SMILES strings were standardized for each molecule. We standardized the structures and ensured that identical structures correspond to identical SMILES strings. This was performed using the CDK framework and our own software. The source codes have been described previously and are available online [7,8].

### 3.2. Data Set Sources and Data Preparation

For the RI_NP and RI_WAX tasks, the NIST 17 database was used as a training set. Molecules not supported by the software used were excluded from the database. In particular, the following molecules were removed: molecules containing 10 or more rings; molecules with a SMILES string representation longer than 250 characters; molecules for which the CDK framework [39] returned an error when processing; molecules containing chemical elements other than B, C, N, O, F, Si, P, S, Cl, Br, and I. The procedure for the initial processing of the data and the compilation of the data set is described in detail in our previous works [7,8]. The NIST database contains many records for each molecule, taken from different sources and obtained under different conditions. During training, these records were considered [7,8] separately, although most of the experimental conditions (in particular, the temperature program) were ignored. In the present study, we used exactly the same computational procedures and data sets as in the previous works [7,8].

After training the machine learning models and performing predictions (for each subset, the model that was trained using all of the other subsets as a training-validation set was used), for each molecule (i.e., for each SMILES string), the median value of all of the predictions (for each model) and the median value of all of the reference values were taken. These median values were considered as the “predicted” and “experimental” values for the given molecule. In the present study, the difference between various non-polar stationary phases (the ”Standard non-polar” and ”Semi-standard non-polar” types in the NIST database) and between various polar ones (the ”Standard polar” type in the NIST database) was ignored. This may lead to some loss in prediction accuracy, but it is not important for the aims of this work. It should be noted that this is exactly the approach that is almost always [6,7,8,31,34,35] used in modern works on predicting gas chromatographic RI using DL.

For HPLC, the open public data set METLIN SMRT [3] was used. The distribution of molecules by RT in this data set is bimodal: some molecules are almost not retained, and the RT of the rest compounds is more than 500 s. All molecules with an RT of less than 500 s were removed. A number of molecules (SMILES strings) occurred more than once; in such cases, one record out of two was randomly removed.

To predict CCS values, a data set (compilation) from the publication [13] was used. The data file (all_training.csv) was downloaded from the corresponding GitHub repository [47]. All records were removed from the file except for the records for [M+H]^+^ ions; those for anions, adducts with sodium, etc., were removed. As in the previous case, a number of molecules (SMILES strings) occurred more than once; in such cases, only one compound was randomly retained.

### 3.3. Clustering and Molecular Similarity Calculation

The following MD were used for clustering: 243 1D and 2D MD generated by the CDK package and 84 functional group counters. The full set of 327 MD is the same as that used in previous studies [7,8]. All MD were scaled so that the values were in the range from 0 to 1. Then, the PCA method was applied and 20 principal components were retained. These 20-dimensional vectors were used for clustering using the *k*-means clustering method. The Euclidean distance was used as the measure. We tried the DBSCAN method [48], but we did not notice a big qualitative difference in the clusters produced; different clustering methods can be used for this task. The clustering result is strongly influenced not only by the method (*k*-means or other), but also by the descriptors used. The number of principal components (and whether this method was used or not) also has an effect. There are many other options: for example, we can consider the Manhattan distance or the Tanimoto metric between binary molecular fingerprints instead of the Euclidean distance. Each of these options will lead to a completely different clustering.

To calculate the molecular similarity, four algorithms were used, based on the Tanimoto similarity between MF, on the cosine similarity between MF, on the Euclidean distance between 20 principal components calculated using MD, and based on MCS. For Tanimoto similarity and cosine similarity, ECFP6 MF calculated using the CDK framework (radius 3, length 8192) were considered. Only additive MF were considered, i.e., MF are a vector of integers rather than bits. This does not affect the calculating of the Tanimoto similarity, but it is important when calculating the cosine similarity. For the Euclidean distance, 20 principal components were used, obtained using the same procedure as for clustering.

The MCS was determined using the rdFMCS.FindMCS method from the RDKit library. The Tanimoto similarity was calculated using the equation *S* = *X*/(*A* + *B − X*), where *X*—sum of the numbers of atoms and bonds in MCS, and *A*, *B*—sums of the numbers of atoms and bonds in two molecules. The following parameters were used: RingMatchesRingOnly = True; CompleteRingsOnly = False, compare bond order. It is important to note that, by default, RDKit does not take into account the aromaticity of atoms when finding MCS; however, we only counted atoms as the same if both were aromatic or both were non-aromatic.

### 3.4. Prediction of Gas Chromatographic Retention Indices

The prediction of gas chromatographic RI for non-polar stationary phases was performed using an ensemble of four models: (i) a 1D convolutional neural network using SMILES strings as input features; (ii) a 2D convolutional neural network using 2D sketches of molecules as input features; (iii) a neural network using MD and MF as input features; and (iv) a GB using MD as input features. The prediction of gas chromatographic RI for polar stationary phases was performed using an ensemble of two models similar to models (i) and (iii) used for the prediction for non-polar stationary phases. All of the models are described in detail in our previous works [7,8]. In the present study, the models and code from the previous works [7,8] were used without any modifications. Information about hyperparameters, such as the number of layers, and the number of channels in convolutional neural networks, is contained in the previous works [7,8]. Supplementary Materials Section S4 contains a brief description of the architecture and hyperparameters.

### 3.5. Prediction of Retention Times in Liquid Chromatography

The prediction of RT in HPLC was performed using graph neural networks. The following atom-wise features were used: (i) one-hot encoded atom type; the considered 25 atom types are provided in the Supplementary Materials Section S5; and (ii) one-hot encoded number (the range from 0 to 4) of hydrogen neighbors. Only heavy atoms were considered as graph vertices. The molecular graph included edges corresponding to bonds, as well as edges corresponding to 1–3 and 1–4 pairs of atoms. The feature vector for each edge had a length of 9. For edges corresponding to bonds, the features included one-hot encoded bond order (the range from 1 to 3), integer encoded bond order, and binary features indicating whether the bond is aromatic and whether it is part of a ring. For edges corresponding to 1–3 and 1–4 atoms, 2 binary features indicating whether a given edge corresponds to a 1–3 or 1–4 pair of atoms were considered. One more binary feature indicates whether the edge matches a bond or a non-bonded pair of atoms.

The MEGNet architecture [49] was used as the architecture of the graph neural network. The implementation (MEGNetConv function) from the GraphNeuralNetworks package [50] (version 0.6.20) for the Julia programming language was used. The following architecture was used. The first dense layer of the neural network (9 input channels and 30 output channels) was applied to each edge, transforming the feature vector of length 9 on each edge into an edgewise vector of length 30. Then, two MEGNetConv layers with 30 and 200 input channels, and 200 and 200 output channels, respectively, were applied to the molecular graph. Each MEGNetConv layer contains neural networks φ_e_, and φ_v_, (a detailed description of the architecture is given in the publication [49]), performing the updating of edge-wise and atom-wise vectors, respectively. In our case, two-layer perceptrons with 200 hidden neurons were used. The second MEGNetConv is used in a residual manner; its output is added to the output of the previous (input) MEGNetConv.

After the second MEGNetConv layer, mean pooling was performed (averaging atom-wise vectors over all atoms). After that, 2 dense layers with 600 and 1 output nodes, respectively, were applied. In all of the layers of the neural network (including those included in MEGNetConv), except for the output layer, the ReLU (rectified linear unit) activation function was used. The MAE was considered as the loss function. The Adam optimizer with a learning rate of 0.0003 and a batch size of 32 was used. The RT values (in seconds) were divided by 1000 before training. In each case, 10% of the molecules randomly selected from the train-validation set were used as the validation set. After each epoch (full run over training set), validation was performed using this set. For further testing, the parameters obtained after the epoch after which the MAE for the validation set was the smallest were used. MAE was also used as a loss function. Training stopped after 100 epochs.

The entire data set was split into 5 subsets; each time, 4 subsets were used as a training-validation set and 1 as a test set. For each test set, training was repeated 5 times (random initialization of weights), and the random split of the training-validation set into training and validation sets was also performed again each time. Thus, a total of 25 graph neural networks were trained.

### 3.6. Prediction of Collision Cross-Sections

The CCS prediction was performed using SVR. The nu-SVR variant of this method with a Gaussian kernel was used. The set of MD used was the same as that used for clustering (see above), but an additional 250 1D and 2D MD computed using RDKit were added (including MD obtained using RDKit’s rdMolDescriptors.MQNs_method). MD with zero variance or matching up to a linear dependence with one of the other MD were removed. The MD values were scaled in such a way that the values ranged from 0 to 1. The CCS (Å^2^) values were divided by 1000 before training.

For each of the 5 test sets, the training was repeated 10 times (random initialization of weights), with 10% of the molecules randomly removed from the training set each time. Thus, a total of 50 models were trained. The hyperparameters for each of the 10 models were different (the same for different test subsets). The hyperparameters *C* (0.13–42), nu (0.46–0.98), gamma (0.0023–0.022), and shrinking (True or False) were subject to change; the tolerance value of 0.001 was used. A satisfactory accuracy was achieved for gamma values of approximately 0.0032·ln(*C*) + 0.014. The exact values of all hyperparameters used in the SVR method are given in the Supplementary Materials Section S6.

## 4. Conclusions

In the present study, models for predicting various mass spectrometry-related properties of molecules were considered. The models considered allow one to estimate retention times and indices, as well as ion collision cross-sections with reasonable accuracy but do not provide any uncertainty estimation. For each specific prediction, there is no assessment of the probability that it is reliable. It was shown that there are at least three ways to estimate the uncertainty: based on the presence in the training set of a molecule similar to the one for which the prediction is made, based on the assignment of the molecule to one of the clusters, and based on the similarity between the predictions of the various models included in the ensemble. It was shown that all three methods allow us to estimate the uncertainty and mark obviously unreliable predictions. In contrast to methods such as Bayesian neural networks, these methods are model-agnostic.

All three methods can be used with ensembles composed of similar models, and with ensembles composed of diverse models, both with neural networks and with other machine learning algorithms. The factors considered can both be used for uncertainty quantification and as the predictors for the classification, to flag predictions that are unsatisfactory with a high probability. The best predictor is the mean absolute deviation of the model predictions from the ensemble prediction. It is demonstrated that these criteria can be used together to assess uncertainty. In this case, the accuracy of marking obviously false predictions increases significantly compared to the use of a single factor. One cannot be sure that the true value is normally distributed because the distribution of prediction errors in the data set is much closer to exponential than to normal. Using this criterion allows for the achieving of values of ROC-AUC (area under the curve) and F1-score measures in the range of 0.690–0.744 and 0.370–0.425, respectively. Using linear regression using all features at once allows for the improving of the values of these measures for 0.727–0.816 and 0.389–0.459, respectively. The average absolute error for those predictions that are marked by the model as unsatisfactory is 2–3 times greater than for other predictions. Clustering-based features are not very accurate if used as the only predictor but can also be used in the model.

Some other methods also used for uncertainty assessment, such as conformal prediction [51,52], were beyond the scope of this work. Of the methods not directly related to chemistry (applicable to any machine learning/deep learning tasks), we included one method that works best, is versatile, model-agnostic, and capable of producing different uncertainty values for different records, that is, ensembles that show the best results for estimating uncertainties among approaches that are not related with chemistry [20,21].

The prediction of mass spectrometry-related properties of molecules using machine learning is of great importance in metabolomics [1,2,3,9], environmental analysis [17], and other fields to confirm the results of mass spectrometric identification. However, when using complex models such as neural networks, in the vast majority of cases, no uncertainty assessment is performed, or the uncertainty assessment is performed by a single method—for example, by comparing the predictions of the models included in the ensemble with each other [6]. Clustering methods are widely used in chemistry [53,54], but we are not aware of any previous consideration of such approaches for estimating uncertainty in predicting mass spectrometry-related properties of molecules. The results obtained in this study can be used in the development of software for non-targeted analyses. When predicting the properties of molecules, such as in chromatographic retention, the use of the considered criteria will allow for the achievement of more meaningful uncertainty estimations. The obtained assessments can be used in scoring candidates for mass spectrometric identification.

## Figures and Tables

**Table 1:** The considered tasks on predicting the mass spectrometry-related properties of molecules.

Task Designation	Task	Models	Ensemble Size	Data Set Size	MAE	MdAE	Units
RI_WAX	RI for polar stationary phases (e.g., DB-WAX)	Two NN * of completely different architectures [8]	2	9408	70.7	28.4	-
RI_NP	RI for non-polar stationary phases (e.g., DB-1, DB-5)	Three NN of completely different architectures and GB [7]	4	88,675	42.1	18.0	-
RT	RT in HPLC (non-retained molecules were excluded)	Five graph convolutional NN of the same architecture	5	77,894	29.1	14.6	Seconds
CCS	CCS ([M+H]^+^ ions)	Ten SVR models with different hyperparameters	10	3532	3.1	1.8	Å^2^

*—neural networks.

**Table 2:** Features for uncertainty estimation used in this work. A detailed description of the algorithms used to calculate the features is contained in the Section 3.

Designation	Description	Range *
*N*_cl_	The number of the cluster to which the molecule belongs	1–20 (categorial feature)
MAE_cl_	Mean absolute prediction error for a given cluster	0–+∞, ↑
MdAE_cl_	Median absolute prediction error for a given cluster	0–+∞, ↑
*D*_cl_	*D*_cl_ = *D*/*D*_mean_, *D* is the Euclidean distance from a given molecule to the centroid of the corresponding cluster, *D*_mean_ is the average distance to the centroid for all molecules included in this cluster	0–+∞, ↑
*S*_max, t_	*S*_max, t_ = max(*S_i_*), where *S_i_*—the Tanimoto similarity between the MF of the *i*-th molecule from the training set and the given molecule	0–1, ↓
*S*_max, mcs_	*S*_max, mcs_ = max(*S_i_*), where *S_i_*—the similarity of the *i*-th molecule from the training set and the given molecule, calculated based on the MCS	0–1, ↓
*S*_max, c_	*S*_max, c_ = max(*S_i_*), where *S_i_*—the cosine similarity between the additive MF of the *i*-th molecule from the training set and the given molecule	0–1, ↓
*S*_max, e_	*S*_max, e_ = min(*S_i_*), where *S_i_*—the Euclide distance between the *i*-th molecule from the training set and the given molecule	0–+∞, ↑
*M*_s1_	The average absolute difference between model predictions and the ensemble prediction	0–+∞, ↑
*M*_s2_	The root mean square difference between model predictions and the ensemble prediction	0–+∞, ↑
*M*_m_	The difference between the smallest and largest predictions of the models	0–+∞, ↑

*—The ↑ sign means that a higher value of this feature indicates a possibly larger error; the ↓ sign means that a higher value of this feature indicates a possibly smaller error.

**Table 3:** The value of *D_t_* (the threshold above which an error is considered unsatisfactory) for different fractions of satisfactory predictions in the data set.

Task Designation	Fraction of Satisfactory Prediction in the Data Set, %			
	80%	85%	90%	95%
RI_WAX	94.5	121.7	170.4	273.5
RI_NP	48.7	62.5	86.4	142.6
RT, seconds	36.3	45.6	62.0	101.9
CCS, Å^2^	4.2	5.2	6.5	9.2

**Table 4:** The ROC-AUC (area under curve) accuracy measure values for distinct prediction tasks and different predictors for uncertainty estimation; a binary classification task is considered, wherein the objective is to predict whether, for a given molecule, the prediction falls within 15% of the least accurate predictions; the *D_t_* values are provided in parenthesis.

Predictor	RI_WAX (121.7)	RI_NP (62.5)	RT (45.6)	CCS (5.2)
MAE_cl_	0.671	0.612	0.573	0.673
MdAE_cl_	0.670	0.635	0.573	0.673
*D*_cl_	0.621	0.592	0.551	0.516
*S*_max, t_	0.720	0.641	0.649	0.654
*S*_max, mcs_	0.634	-	-	-
*S*_max, c_	0.686	0.615	0.623	0.635
*S*_max, e_	0.689	0.657	0.614	0.707
*M*_s1_	0.744	0.742	0.690	0.709
*M*_s2_	0.744	0.734	0.691	0.711
Logistic regression	0.816	0.778	0.727	0.741
AutoML 1	0.819	0.801	0.733	0.752

**Table 5:** Coefficients in the logistic regression equation for uncertainty estimation; standard deviation is shown in parentheses.

Feature	RI_WAX	RI_NP	RT	CCS
MAE_cl_	−0.0010 (0.0012)	0.0042 (0.0009)	0.0111 (0.0028)	−0.0111 (0.0171)
*D*_cl_	0.2750 (0.1045)	0.1260 (0.0310)	−0.0536 (0.0542)	−0.6967 (0.1722)
*S*_max, t_	−3.6407 (0.2441)	−2.8190 (0.0701)	−2.9727 (0.0933)	−1.8850 (0.3406)
*S*_max, e_	0.8981 (0.2034)	2.1392 (0.1110)	0.6423 (0.0755)	1.2584 (0.3256)
*M*_s1_	0.0152 (0.0009)	0.0221 (0.0004)	0.0373 (0.0011)	0.5087 (0.0874)
Ensemble prediction divided by 1000	0.8935 (0.0745)	0.3903 (0.0223)	1.9997 (0.0686)	4.7782 (1.4209)

**Table 6:** The accuracy of logistic regression, which predicts whether predictions are unsatisfactory (positive = unsatisfactory).

Measure	RI_WAX	RI_NP	RT	CCS
True positives	1030	8924	7104	342
True negatives	5954	56,715	48,505	2135
False positives	2058	18,654	17,692	868
False negatives	366	4382	4593	187
F1-score	0.459	0.437	0.389	0.393
Precision	0.334	0.324	0.286	0.283
Recall	0.738	0.671	0.607	0.647
Accuracy	0.742	0.740	0.714	0.701

**Table 7:** The number of predictions that are labeled as satisfactory and unsatisfactory by logistic regression (*N*_sat_ and *N*_unsat_, respectively); mean absolute error for predictions labeled as satisfactory and unsatisfactory by logistic regression, as well as for all predictions (MAE_sat_, MAE_unsat_, and MAE_all_, respectively); the threshold value of 0.15 is the same as for Table 6.

Task	*N*_sat_	*N*_unsat_	MAE_sat_	MAE_unsat_	MAE_all_
RI_WAX	6320	3088	38.5	136.5	70.7
RI_NP	61,097	27,578	27.7	74.0	42.1
RT	53,098	24,796	21.7	44.8	29.0
CCS	2322	1210	2.1	4.9	3.1

**Table 8:** The accuracy of automated machine learning, which predicts whether predictions are unsatisfactory (positive = unsatisfactory); standard deviations over five cross-validation subsets are given in parenthesis.

Measure	RI_WAX	RI_NP	RT	CCS
ROC-AUC	0.819 (0.010)	0.801 (0.004)	0.733 (0.005)	0.752 (0.034)
F1-score	0.484 (0.020)	0.475 (0.005)	0.419 (0.011)	0.428 (0.032)
Precision	0.383 (0.030)	0.396 (0.006)	0.376 (0.016)	0.355 (0.035)
Recall	0.664 (0.042)	0.595 (0.013)	0.474 (0.017)	0.545 (0.056)
Accuracy	0.790 (0.018)	0.803 (0.004)	0.803 (0.008)	0.783 (0.017)

## Data Availability

Data sets used for CCS and RT tasks are publicly available; the corresponding references are provided in the text. The source code for computing features that characterize uncertainty is available in the repository: https://github.com/mtshn/uncertaintychem, accessed on 4 November 2024.

## Supplementary Materials

The following supporting information can be downloaded at: https://www.mdpi.com/article/10.3390/ijms252313077/s1.
