# Supplementary material for: Uncertainty Quantification and Flagging of Unreliable Predictions in Predicting Mass Spectrometry-Related Properties of Small Molecules Using Machine Learning
Source: Int J Mol Sci. 2024 Dec 5;25(23):13077. doi: 10.3390/ijms252313077 (PMC11641629; doi:10.3390/ijms252313077)
Supplement: Supplementary file 1 [file ijms-25-13077-s001.zip › ijms-3331860-supplementary.pdf]

Supplementary Material for the article “Uncertainty quantification and flagging of unreliable predictions in predicting mass spectrometry-related properties of small molecules using machine learning”

Dmitriy D. Matyushin, Ivan A. Burov, Anastasia Yu. Sholokhova*

A.N. Frumkin Institute of Physical Chemistry and Electrochemistry, Russian Academy of Sciences, 31 Leninsky Prospect, Moscow, GSP-1, 119071, Russia

*Corresponding author

E-mail address: shonastya@yandex.ru (Anastasia Yu. Sholokhova)

S1. The ROC-AUC (area under curve) accuracy measure values for distinct prediction tasks and different predictors for uncertainty estimation

A binary classification task is considered, wherein the objective is to predict whether for a given molecule the prediction falls within the 15% least accurate predictions. The F1 values are given for the threshold value for which they are maximum.

Predictor	RI_WAX (121.7)	RI_NP (62.5)	RT (45.6)	CCS (5.2)
MAE_{cl}	0.329	0.310	0.277	0.331
$MdAE_{cl}$	0.329	0.323	0.275	0.331
D_{cl}	0.302	0.281	0.264	0.263
$S_{max, t}$	0.372	0.324	0.339	0.325
$S_{max, c}$	0.344	0.297	0.310	0.311
$S_{max, e}$	0.362	0.352	0.307	0.374
M_{s1}	0.425	0.415	0.370	0.387
M_{s2}	0.425	0.403	0.371	0.388
Logistic regression	0.459*	0.437*	0.389*	0.393*
AutoML 1	0.484	0.475	0.419	0.428

* - threshold value 0.15

S2. Recall-precision curves for different tasks and classifiers

Data are given for 85% satisfactory predictions in the data set.

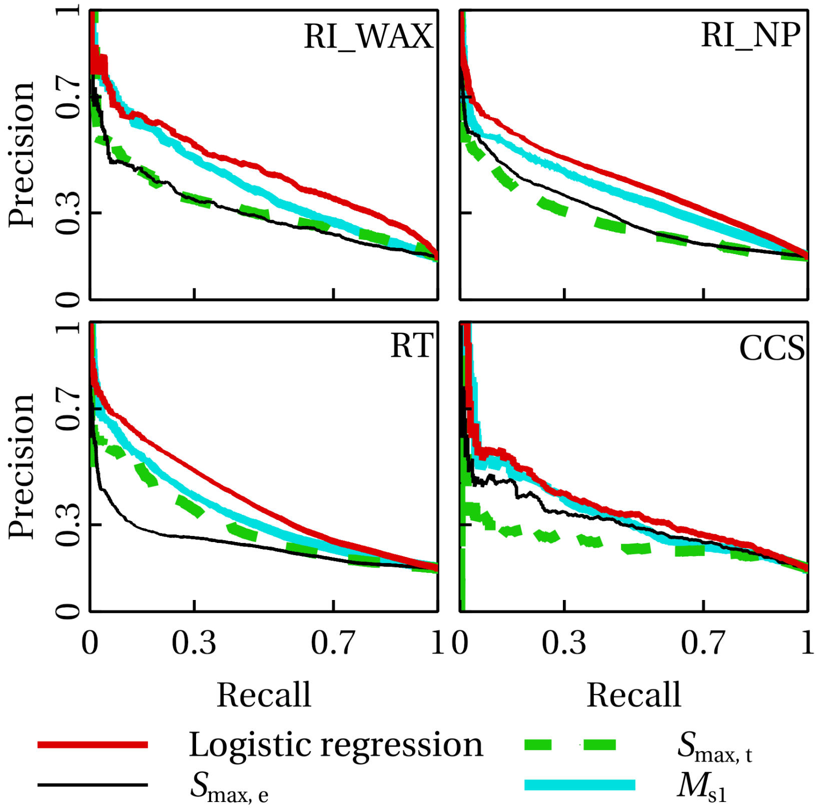

S3. Dependence of the relative prediction error on the features characterizing the accuracy of the prediction

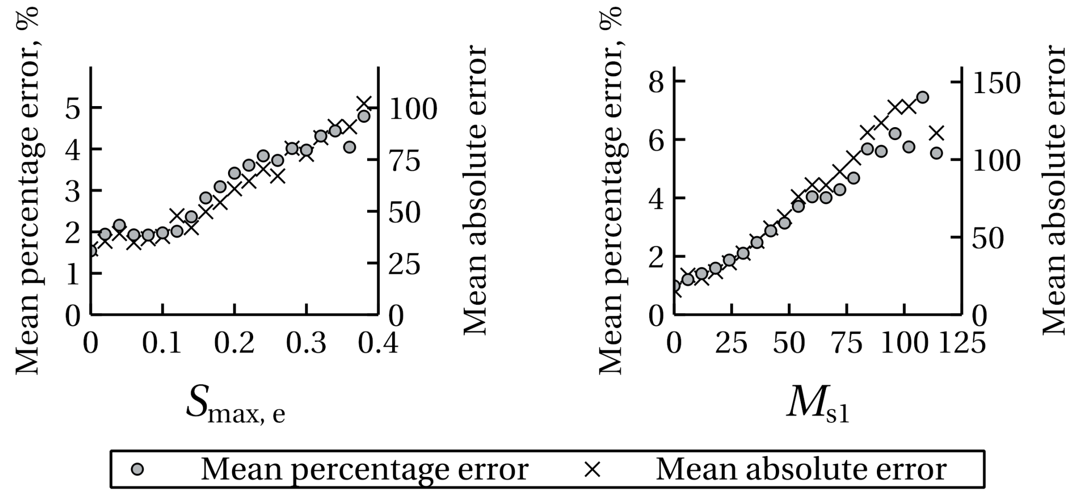

S4. Hyperparameters of models for predicting gas chromatographic retention indices

CNN1D
Input channels: 36 (SMILES strings, symbols were one-hot encoded); the number of 1D convolutional layers: 2; kernel: 6; stride: 1; output channels: 300; pooling: MAX; dense layers: 2 (600 and 1 output nodes)

CNN2D
Input channels: 29 (2D sketch of molecule with atom types specified); number of 2D convolutional layers: 3; kernel: 4*4; stride = 1; output channels: 50, 300, 300; first 2 2D convolutional layers are followed by MAX-pooling subsampling layers (2*2); pooling: MAX; dense layers: 2 (600 and 1 output nodes)

MLP
Two-input neural network with subsequent concatenation.

First input: molecular descriptors and stationary phase information, activation function after the input layer: TANH, activation function after the second layer: ReLU, two layers, 300 output nodes.

Second input: additive molecular fingerprints (ECFP4, length = 1024); 5 dense layers with 1200 output nodes, on all layers except the input dropout = 0.05; the second, third, fourth, and fifth layers are combined into 2 residual blocks with 2 layers each.

After concatenation, two dense layers, 600 and 1 output nodes.

XGBoost
eta = 0.05, gamma = 0.05, lambda = 0.05, max_depth = 21, min_child_weight = 21, subsample = 0.5, colsample_bytree = 0.5, n_estimators = 800

In convolutional neural networks, one-hot encoded information about the stationary phase type is concatenated with the pooling output. In other cases, with molecular descriptors. Neural networks were trained only for non-polar stationary phases at the first stage. Transfer learning for polar stationary phases was made in the second stage. Both polar and non-polar stationary phases were not present in the data set at the same time. For polar stationary phases, only CNN1D and MLP were used. There was no activation function after the output layers. Unless otherwise stated, the ReLU activation function was used everywhere else. Loss function: MAE (all neural networks), RMSE (XGBoost).

A detailed description of the architectures (with figures), all hyperparameters and the training procedure are given in the following articles (open access):
Matyushin DD, Buryak AK. Gas chromatographic retention index prediction using multimodal machine learning. Ieee Access. 2020 Dec 15;8:223140-55. 10.1109/ACCESS.2020.3045047
Matyushin DD, Sholokhova AY, Buryak AK. Deep learning based prediction of gas chromatographic retention indices for a wide variety of polar and mid-polar liquid stationary phases. International journal of molecular sciences. 2021 Aug 25;22(17):9194. 10.3390/ijms22179194

S5. Considered types of atoms for graph neural network

```
String[] atomTypes = new String[] { "B", "C", "F", "Cl", "I", "S.planar3",
"C.sp2", "C.sp3", "N", "O", "P", "N.nitro", "S", "X", "S.3", "O.planar3",
"N.planar3", "Br", "N.amide", "N.sp2", "Si", "N.sp3", "C.sp", "O.sp3", "O.sp2" };
```

The atom types "C", "O", etc. should be understood as "other", not fitting into more specific types.

S6. Hyperparameters of models for prediction predicting collision cross-sections

nu-SVR with Gaussian kernel

C: 0.3738255 nu: 0.7584833 gamma: 0.019689038 shrinking: true RMSE: 4.839763 MAE: 3.0028067
C: 0.7977819 nu: 0.53467625 gamma: 0.020517752 shrinking: false RMSE: 4.850297 MAE: 3.026735
C: 1.5623616 nu: 0.46790478 gamma: 0.016570907 shrinking: true RMSE: 4.87208 MAE: 3.053219
C: 1.2482815 nu: 0.57479703 gamma: 0.009520819 shrinking: true RMSE: 4.858917 MAE: 3.077646
C: 8.786146 nu: 0.5814111 gamma: 0.0059047374 shrinking: true RMSE: 4.9055176 MAE: 3.084831
C: 0.15018477 nu: 0.67823684 gamma: 0.017828526 shrinking: false RMSE: 4.8587766 MAE: 3.0965083
C: 41.69839 nu: 0.59048045 gamma: 0.0023419203 shrinking: true RMSE: 4.9265375 MAE: 3.1041965
C: 8.676583 nu: 0.7513188 gamma: 0.0034053398 shrinking: true RMSE: 4.986075 MAE: 3.1169972
C: 0.15323155 nu: 0.6641317 gamma: 0.015355766 shrinking: true RMSE: 4.869419 MAE: 3.122323
C: 0.13144547 nu: 0.98174363 gamma: 0.02155845 shrinking: false RMSE: 4.93376 MAE: 3.1254199
